# Clinical and genetic features of Fanconi anemia associated with a variant of *FANCA* gene: Case report and literature review

**DOI:** 10.1097/MD.0000000000039358

**Published:** 2024-09-06

**Authors:** Lin Zhong, Wenhua Zhang, Kaihui Zhang, Chan Li, Xiao Mu, Yan Chu, Zhongtao Gai, Haiyun Wei

**Affiliations:** a Pediatric Research Institute, Children’s Hospital Affiliated to Shandong University, Jinan Children’s Hospital, Jinan, Shandong, China; b Department of Medical Ultrasound, the First Affiliated Hospital of Shandong First Medical University, Shandong Provincial Qianfoshan Hospital, Jinan, Shandong, China; c Prenatal Diagnosis Center of The Tenth Affiliated Hospital, Southern Medical University, Dongguan People’s Hospital, Dongguan Key Laboratory of Major Diseases in Obstetrics and Gynecology, Dongguan, Guangdong, China; d Burn and Plastic Surgery Department, the 80th Army Group Hospital, Weifang, Shandong, China.

**Keywords:** allogeneic hematopoietic cell transplantation, *FANCA*, Fanconi anemia, gene mutation, prenatal diagnosis

## Abstract

**Rationale:**

Fanconi anemia (FA) is a hereditary disease caused by mutations in the genes involved in the DNA damage repair pathway. The *FANCA* gene is the most commonly pathogenic gene, accounting for more than 60% of all causative genes.

**Patient concerns:**

The clinical case is a 3-year-old boy showed mild anemia and scattered bleeding spots the size of a needle tip all over his body.

**Diagnoses:**

Compound heterozygous mutation was identified in the *FANCA* gene in the FA case: c.1A > T from the father in exon 1; the deletion of chr16: 89857810-89858476 (exon13-14 del) from the mother; finally, the patient was diagnosed as Fanconi anemia.

**Intervention:**

After diagnosis, the child received chemotherapy (Ara-C + Flu + Cy + ATG). Then, the hematopoietic stem cell transplantation and unrelated umbilical cord blood transfusion were performed.

**Outcomes:**

The child is recovering well and is in regular follow-up.

**Conclusion and Lessons:**

The discovery of new mutations in the *FANCA* gene enriches the genetic profile of FA and helps clinicians to further understand this disease and guide genetic counseling and prenatal diagnosis. Whole-exome sequencing is a powerful tool for diagnosing FA.

## 1. Introduction

Fanconi anemia (FA) is a genomic instability syndrome that is inherited in 3 ways: X-linked recessive (XR), autosomal recessive (AR), and autosomal dominant (AD).^[1]^ It mainly manifests as bone marrow failure (BMF), increased susceptibility to cancer, and congenital developmental anomalies; approximately 75% of patients have different degrees of physical characteristics, including microcephaly, short stature, eye abnormalities, urinary system malformations, skeletal deformities, and skin pigmentation.^[2]^ These features can be used for the early identification and diagnosis of FA. The Fanconi pathway plays an important role in detecting and repairing DNA damage. At least 23 genes are involved in this pathway, and mutations in any of these genes can affect DNA repair, causing the accumulation of DNA fragmentation, genomic instability, and the occurrence of FA.^[3]^ Among these 23 genes, *FANCR* is inherited in the AD manner, *FANCB* is inherited in the XR manner, and the rest are all inherited in the AR manner.^[4]^ *FANCA* is the most commonly (60%–70%) mutated gene in FA. It is located in 16q24.3 and contains 43 exons spanning approximately 80 kb, with the exome size ranging from 34 bp to 188 bp. The protein encoded by *FANCA* is involved in the composition of the nuclear multiprotein core complex, which triggers a series of reactions during the S phase of the growth cycle and after exposure to DNA cross-linking, and in DNA damage repair.^[5,6]^

Only a few case reports of prenatal diagnosis related to FA in China have been published. In this study, we discovered a new mutation of the *FANCA* gene. We focused on the clinical manifestations, diagnosis, and treatment of this case. The findings will be useful for the early identification and diagnosis of FA and could guide the prenatal diagnosis of the next child and enrich the gene profile related to this disease.

## 2. Materials and methods

### 2.1. Compliance with ethical standards

The materials and methods of this study were ethical. This work was permitted by the Medical Ethics Committee of Dongguan People’s Hospital (DGRM-20230327). Before the clinical and laboratory tests, the patient’s parents had already given written informed consent, and all procedures conformed to the standards of the Declaration of Helsinki.

### 2.2. Patients

The patient was questioned in detail about his clinical presentation and given a thorough physical examination. For genetic analysis, the medical history of the patient and his family was also collected at the same time.

### 2.3. Routine tests

Blood was collected from the child and then routinely subjected to laboratory biochemical and flow cytometry examinations. Briefly, we collected 2 ml of bone marrow fluid for routine bone marrow smear cytology, and a small portion of the bone marrow was collected using a bone marrow biopsy needle.

### 2.4. Chromosome karyotyping

Briefly, 2 ml of peripheral venous blood was collected under aseptic conditions with heparin anticoagulation for chromosome karyotype analysis. G-banding karyotype analysis was performed regularly. Peripheral blood lymphocytes were cultured at 37°C for 72 hours, harvested, prepared, subjected to G-banding, counted, and analyzed using a Lycra automatic genetic workstation. Twenty divisions were counted, and 5 karyotypes were analyzed.

### 2.5. Targeted captured sequencing

Briefly, 2 ml of peripheral blood was extracted from the child and his parents and then added with ethylene diamine tetraacetic acid anticoagulation. Genomic DNA was extracted, and an exon capture kit (item No. 1080585, IDT, Germany) was used to target the DNA exons of the child and his parents. Whole-exome sequencing (WES) was performed using Illumina NovaSeq 5000 high-throughput sequencing platform (Illumina, USA). Then, use NextGene V2.3.4 software to compare the sequencing data with the human genome hg19 reference sequence, which is provided by the University of California Santa Cruz (https://genome.ucsc.edu/) database. The average exome coverage was over 95% (>10x coverage, average depth over 100x). The variants were filtered following strict screening criteria, and those leading to nonsense, frameshift, missense, start codon loss, and typical splice site change were chosen.

### 2.6. Verification of genetic variations

Sanger sequencing and quantitative Real-time PCR (qPCR) were used to verify genetic variants. PCR amplification of DNA at the mutation site region was conducted using specific primers under the following reaction conditions: 95°C predenaturation for 5 minutes and 30 cycles of amplification with 95°C denaturation for 30 seconds, 57°C annealing for 30 seconds and 72°C extension for 30 seconds. The PCR amplification products of the children and their parents were sequenced by Sanger at 72°C for 7 minutes. qPCR primers were designed (see Table S1, Supplemental Digital Content, http://links.lww.com/MD/N451), and qPCR verification was used for the heterozygous deletion of maternal origin. Peripheral blood RNA was extracted from the child and his parents, and cDNA synthesis was performed following the instructions of the iScript Synthesis Kit and the iTaq Universal SYBR Green Supermix. The reagent instructions were adopted for qPCR. Three replicates were set for all reactions and placed in the qPCR instrument, and qPCR amplification was carried out according to the set program.

## 3. Results

### 3.1. Clinical characteristics and laboratory tests

**Figure 1.**
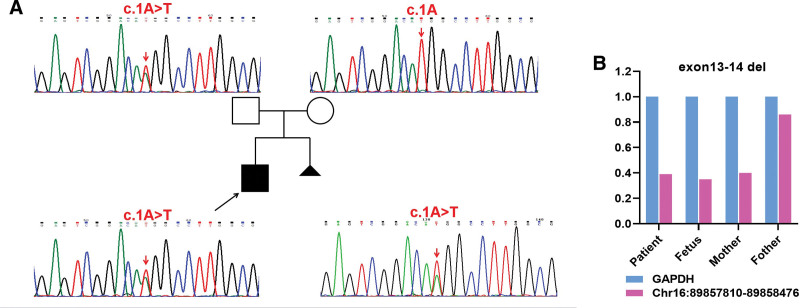
Identification results of WES, Sanger sequencing and qPCR in this case. (A) Genetic pedigree of the disease in this case. The proband and fetus carried the mutation c.1A > T from the mother. (B) Deletion mutation (exon13-14 del) in the *FANCA* gene inherited from the mother (chr16:89857810-89858476) in this case. The gross deletion was tested by SYBR qPCR, and the Y-coordinate’s number represents the ratio of the deletion region to the copy of the glyceraldehyde-3-phosphate dehydrogenase gene. Normal copy numbers are generally 0.7 < 2^−ΔΔCt^ <1.3, 0.3 < 2^−ΔΔCt^ <0.6 is heterozygous loss. P: Proband; F: Father; M: Mother. qPCR = quantitative Real-time PCR, WES = whole-exome sequencing.

The proband, a 3-year-old boy, was admitted to Dongguan People’s Hospital in August 2018 due to “pale face, ecchymosis and petechiae all over the body.” The child was the mother’s first fetus and first child. The proband’s mother had no abnormalities during pregnancy and no history of abnormal delivery. His physical development was slightly behind that of children of the same age. At the time of examination, the child had mild anemia and scattered bleeding spots the size of a needle tip all over his body, and no other evident signs were found. Routine blood test showed neutrophils were 0.3 × 10^9^/L (Ref: 2–7.5 × 10^9^/L), hemoglobin was 68 g/L (Ref:120–160 g/L), and platelet was 9 × 10^9^/L (Ref:100–300 × 10^9^/L). The reticulocyte count was 0.015% (Ref:0.5%–1.5%), the absolute value of reticulocytes was 45 × 10^9^/L (Ref:24–84 × 10^9^/L), hemoglobin was HbF 11.8% (Ref:0%–2.5%), HbH inclusion body was negative (Ref:0%–5%), HbA2 2.3% (Ref:1.4%–3.6%) and HbA 85.9% (Ref: 96.5%–100%). No significant abnormality was found in the results of fluorescence in situ hybridization. Bone marrow cytology showed that the bone marrow hyperplasia was active, granulocyte/erythrocyte = 14:1 (Ref:[2–4]:1), the proportion of lymphocytes was increased and accounted for 77.5%, no megakaryocytes were found in the whole bone marrow smear, the platelets were rare and cells with unknown classification accounted for 1.0%. Bone marrow biopsy showed that the proportion of erythrocytes increased slightly, the erythroid series decreased slightly and the erythrocytes were mainly in the middle and late stages. A decrease in lobulated nuclear cells and a slight increase in monocytes were observed. Apoptosis was visible, and megakaryocytes were not found in the whole bone marrow smear. Flow cytometry showed the percentage of monocyte CD59+ at 95.48%, granulocyte CD59 at 97.27%, erythrocyte CD59+ at 99.50%, monocyte CD55+ at 100.0%, granulocyte CD55+ at 99.6% and erythrocyte CD55+ at 98.41%. The chromosome break test was negative. Except for the above case, this family had no history of related diseases or other malignant diseases (see Fig. 1A).

### 3.2. Chromosome karyotyping

The chromosomal karyotype of the child showed few cells, and no nucleomorph was observed.

### 3.3. Whole exon gene testing, Sanger sequencing and qPCR

The sequencing results of the proband showed that the coverage of the target genes with a sequencing depth greater than 20 × was more than 97.34% and the total coverage was more than 99.40%, indicating that the data quality met the standard requirements. Two variants were found in the *FANCA* gene of the proband. The chromosomal location of the first mutation was located in chr16: 89857810-89858476, showing that the 13th and 14th exons of this location were deleted (exon13-14 del) and originated from the mother. The second mutation is the first codon mutation that results in a change in amino acid composition (c.1A > T) inherited from his father. The two mutations are highly associated with FA, complementary group A (OMIM: 227650; AR). We then performed Sanger sequencing verification based on the gene variant with the high probability of pathogenicity and found that the proband and his father had the same point mutation (c.1A > T) at this location (Fig. 1A). Also, qPCR results showed that the deletion mutation was originated from his mother(Fig. 1B).

### 3.4. Pathogenicity analysis of the gene mutated sites

The detected variants were subjected to pathogenicity analysis in accordance with the American College of Medical Genetics and Genomics guidelines (2015). Variant 1, exon13-14 del, is a novel variant with a possible loss of gene function due to the Loss of Function variant and therefore named as hyperpathogenic (pathogenic very strong [PVS1]). None of the variants were found in the normal control population database, so the evidence of causative disease was classified as pathogenic moderate 2 (PM2). In this recessive genetic disorder, a pathogenic variant was detected at the trans location, thus classifying the evidence of pathogenicity as pathogenic moderate 3 (PM3). Based on the above factors (PVS1 + PM2 + PM3), we listed the variant as the causative gene. The variant, c.1A > T, is also a causative gene with super pathogenicity (PVS1), and its variant type is the same as the identified causative variant. This evidence of pathogenicity was classified as PS1. The Minor Allele Frequency of this variant in the dbSNP database was 0.000024 (<0.005), which is a low-frequency variant, so the evidence of pathogenicity was classified as PM2 level.

Based on the clinical symptoms (pancytopenia, developmental delay) and genetic test results, we finally diagnosed the child with FA, complementary group A (OMIM: 227650).

### 3.5. Diagnosis, treatment, and follow-up

Initially, the child was diagnosed with aplastic anemia (AA) without genetic testing. Therefore, he was treated as AA with oral cyclosporine and other drugs, but the efficacy was poor and the quantities of red blood cells and platelets were still low. In January 2019, WES was performed, and 2 *FANCA* gene mutations were found. Based on the child’s clinical symptoms (pancytopenia, developmental delay) and genetic result, we finally diagnosed him with FA, complementary group A (OMIM: 227650). After an accurate diagnosis, the child received chemotherapy (Ara-C + Flu + Cy +  antithymocyte globulin [ATG]). Hematopoietic stem cell transplantation and unrelated umbilical cord blood transfusion were then performed as planned on October 11, 2019. After transplantation, anti-infective medications such as methylprednisolone and tacrolimus, anti-immune drug teicoplanin, and azithromycin were given (Dongguan Taixin Hospital). At present, the child is recovering well, and the blood cell count was normal, including neutrophils, hemoglobin, and platelets.

The proband’s mother became pregnant again in 2023. At 17 + weeks of pregnancy, an ultrasound examination showed no evident abnormalities. Thus, we performed prenatal genetic diagnosis for the *FANCA* gene compound heterozygous variant locus of the proband, and the results showed that the chromosome and microarray results were normal. However, WES on the amniotic fluid obtained by amniocentesis showed that the fetus had the 2 suspected pathogenic gene mutations, which was consistent with the type of proband’s mutation. Hence, intrauterine abortion was performed on the mother at 17+ weeks of pregnancy.

## 4. Discussion

FA is the most common congenital BMF disease, with an estimated incidence of 1 in 130,000 in the population; it is characterized by sensitivity to DNA cross-linkers, progressive BMF, congenital developmental abnormalities, and increased susceptibility to cancer.^[1]^ The clinical manifestations of FA are different, and affected individuals may have one clinical manifestation at least, such as hypoplasia, pancytopenia, skin pigmentation (café-au-lait spots), microcephaly, radial dysplasia, and genitourinary tract malformations (including renal absence and cryptorchidism).^[7]^ The patients in this article present with pancytopenia and growth retardation, which is consistent with the clinical presentation of the disease. *FANCA* gene mutation is the most common in FA, and its biallelic mutation accounts for more than 60% of the total cases. Although heterozygous *FANCA* gene mutation does not definitively cause FA, it may cause ovarian insufficiency in women and thus affect their reproductive ability.^[8]^ Additionally, heterozygous mutations in the *FANCA* gene increase cancer susceptibility in a sporadic manner, which is associated with a variety of tumor diseases such as gastric cancer, prostate cancer, and colorectal cancer.^[9]^

AA is one of the major complications of FA,^[10]^ and many patients present with AA-related manifestations, such as paleness and ecchymosis, and petechiae all over their body.^[11]^ Therefore, accurately distinguishing between FA and AA is quite important for the early diagnosis and therapy of FA. The chromosomal break test is the gold standard test for diagnosing FA, and the peripheral blood lymphocytes of patients with FA are highly sensitive to DNA cross-linkers (such as mitomycin C [MMC] and diepoxybutane [DEB]). First, the patient’s peripheral blood is placed in DEB or MMC, and chromosomal instability data are analyzed and calculated; if the chromosome breaks and radial forms increase, the result is positive and further confirms the diagnosis of FA.^[12]^ However, the specificity of this diagnosis is not 100%, and false-negative and false-positive results still exist.^[13]^ Therefore, molecular testing still plays an important role in FA, especially in typing, predicting prognosis, genetic counseling, and prenatal diagnosis. Single-gene testing can be used for specific genes (e.g., the *FANCA* gene with the highest mutation rate), and multi-gene panels can be applied when single-gene testing is ineffective or comprehensive genomic testing is necessary.^[6]^ Designing specific genomic assays for different patients is beneficial to improve the efficiency and sensitivity of FA diagnosis.

Allogeneic hematopoietic cell transplantation (HCT) is the only treatment for hematologic manifestations (e.g., BMF, leukemia, and myelodysplastic syndrome) in FA.^[14]^ Given that patients with FA are highly sensitive to radiotherapy and chemotherapy due to their own DNA repair defects, HCT conditioning regimens specific for them must contain significantly reduced chemoradiotherapy doses to avoid excessive toxicity accumulation.^[15]^ Over the past few decades, the survival rate of patients with FA after HCT has improved significantly due to advancements in prechemotherapy regimens, especially the use of fludarabine (Flu).^[16]^ Regarding the pretreatment regimen of HCT, the current national standards are not uniform. Up to now, cyclophosphamide/fludarabine/thymoglobulin(Cy/Flu/ATG) is the most commonly used regimen. The addition of busulfan (Bu) depends on the protocol of local institutions.^[14]^ In 2019, Mehta et al^[15]^ conducted the first prospective pharmacokinetic study of prospective Bu in patients with FA receiving HCT and determined the optimal target Css level of Bu in HCT in these patients (≤350 ng/ml). Their results can guide the precise use of Bu in HCT in the future to stabilize donor implantation and avoid excessive toxicity. Although the early mortality rate of patients with FA receiving HCT can be significantly improved, the risk of developing solid tumors remains high; even chronic graft-versus-host disease can increase the risk of solid tumors, particularly squamous cell carcinoma of the head and neck.^[17,18]^ Therefore, a treatment that can improve early and long-term outcomes must be urgently developed. At present, gene therapy (GT) has gradually become a hot spot in FA treatment. For the first time, a team in Spain demonstrated that lentivirus-mediated GT can successfully restore the transcriptional program of hematopoietic stem cells and progenitor cells (HSPCs) in patients with FA, bringing them close to expressing healthy HSPCs.^[19]^ Siegner et al^[20]^ found that editing mutations in the *FANCA* gene using an adenine base editor can lead to the re-expression of *FANCA* proteins and reactivation of the FA pathway in edited lymphoblast-like cell lines with significant value-added advantages over unedited cells. These phenomena were also demonstrated in HSPCs. This study suggested that adenine base editor has great potential in restoring HSPC function in patients with FA. However, these trials have a short duration, and long-term follow-up studies are lacking. Whether these therapies can improve the long-term prognosis of patients with FA remains unclear, and further studies are still needed for confirmation.

At present, the method of collecting maternal amniotic fluid or chorionic amniotic membrane samples by invasive means (such as amniocentesis) for WES has gradually become a powerful tool for prenatal diagnosis. It can detect mutations in the *FANCA* gene such as in this case and find variants in *COL2A1, KMT2D, THOC6,* and other genes involved in Stickler syndrome, Kabuki syndrome, and Beaulieu–Boycott–Innes syndrome, greatly contributing to the development of genetic counseling.^[21]^ Scientists are currently trying to find a noninvasive method to replace the traditional invasive way of collecting samples to avoid the risk of miscarriage due to the procedure.^[22]^

In conclusion, the proband in this study was stunted and had pancytopenia, basically matching the phenotype of FA. We discovered a new type of *FANCA* gene mutation through WES. The findings further expand the genetic profile of the disease and help in studying the genotype-phenotype relevance and prenatal diagnosis of FA.

## 5. Limitation

Currently, hematopoietic stem cell transplantation is an effective treatment for FA, but there is a risk of relapse, requiring long-term observation and follow-up. The follow-up period for the patient is too short, and the long-term prognosis requires further tracking and observation. Further experimental work to verify the pathogenic for the 2 mutations is necessary.

## Acknowledgments

The authors gratefully acknowledge the participation and cooperation of patients in this study.

## Author contributions

**Data curation:** Lin Zhong, Wenhua Zhang

**Methodology:** Lin Zhong, Zhongtao Gai

**Writing – original draft:** Lin Zhong

**Formal analysis:** Kaihui Zhang

**Writing – review & editing:** Kaihui Zhang

**Investigation:** Chan Li, Xiao Mu, Yan Chu

**Supervision:** Haiyun Wei

## Supplementary Material


