# Supplementary material for: Clinical and genetic features of Fanconi anemia associated with a variant of FANCA gene: Case report and literature review
Source: Medicine (Baltimore). 2024 Sep 6;103(36):e39358. doi: 10.1097/MD.0000000000039358 (PMC12431735; doi:10.1097/MD.0000000000039358)
Supplement: Supplementary file 1 [file medi-103-e39358-s001.docx]

**Supplementary Table 1.** Primers of qPCR for *FANCA* gene

| **Gene** | **Forward** | | **Reverse** | |  | |
| --- | --- | --- | --- | --- | --- | --- |
| **FANCA** | ACAACCAGGAACGCAGTGA | | TTGCCTTGCTGTGACTATGTC | |  | |
| **GAPDH** | GTCTCCTCTGACTTCAACAGCG |  | ACCACCCTGTTGCTGTAGCCAA |  |  |  |
